# Homeostatic Plasticity Mediated by Rod-Cone Gap Junction Coupling in Retinal Degenerative Dystrophic RCS Rats

**DOI:** 10.3389/fncel.2017.00098

**Published:** 2017-04-20

**Authors:** Baoke Hou, Yan Fu, Chuanhuang Weng, Weiping Liu, Congjian Zhao, Zheng Qin Yin

**Affiliations:** ^1^Southwest Hospital/Southwest Eye Hospital, Third Military Medical UniversityChongqing, China; ^2^Department of Ophthalmology, Chinese PLA General HospitalBeijing, China; ^3^Key Lab of Visual Damage and Regeneration and Restoration of ChongqingChongqing, China

**Keywords:** gap junctions, retinal pigmentosa, synaptic plasticity, melatonin, AANAT

## Abstract

Rod-cone gap junctions open at night to allow rod signals to pass to cones and activate the cone-bipolar pathway. This enhances the ability to detect large, dim objects at night. This electrical synaptic switch is governed by the circadian clock and represents a novel form of homeostatic plasticity that regulates retinal excitability according to network activity. We used tracer labeling and ERG recording in the retinae of control and retinal degenerative dystrophic RCS rats. We found that in the control animals, rod-cone gap junction coupling was regulated by the circadian clock via the modulation of the phosphorylation of the melatonin synthetic enzyme arylalkylamine N-acetyltransferase (AANAT). However, in dystrophic RCS rats, AANAT was constitutively phosphorylated, causing rod-cone gap junctions to remain open. A further b/a-wave ratio analysis revealed that dystrophic RCS rats had stronger synaptic strength between photoreceptors and bipolar cells, possibly because rod-cone gap junctions remained open. This was despite the fact that a decrease was observed in the amplitude of both a- and b-waves as a result of the progressive loss of rods during early degenerative stages. These results suggest that electric synaptic strength is increased during the day to allow cone signals to pass to the remaining rods and to be propagated to rod bipolar cells, thereby partially compensating for the weak visual input caused by the loss of rods.

## Introduction

The retinal circadian clock governs the extent and strength of rod-cone gap junction coupling in the retinae of vertebrates, including macaques, mice, goldfish, and zebrafish (Ribelayga et al., 2002; Völgyi et al., 2013; Li et al., 2014). This functional switch allows cones to receive very dim light signals from rods at night but not during the day (Ribelayga et al., 2008). The process of increasing rod-cone gap junction coupling may provide vertebrates with the ability to detect large, dim objects. The mechanism underlying the regulation of rod-cone synapses resembles homeostatic plasticity but involves electrical synapses and circadian clock-regulated effects on the compensatory shaping of rods (Curti and O'Brien, 2016).

The Royal College of Surgeons (RCS) rat is a good animal model for studying the early onset of retinal degenerative diseases (Sauvé et al., 2001). In this rat, a substantial loss of rod photoreceptors occurs several months before the loss of cones, and the combination of these events results in eventual blindness (Girman et al., 2005). Retinal degeneration is initiated in this animal model during late retinal synaptic functional maturation (Van Den Berghe et al., 2004). The synaptic connections and synaptic strength between photoreceptors and bipolar cells are still being refined during late maturation, after the eyes have opened. In RCS mice, a malfunctional RPE results in rod cell death (D'Orazi et al., 2014). Gap junctions but not rod-cone coupling via gap junctions have been observed in rat photoreceptors (Guo et al., 2014). Presumably, during the progressive loss of rods in dystrophic RCS rats, the loss of night vision would be partially compensated if signaling by the remaining rods was capable of reaching the cones via gap junction couplings. Here, we investigated whether gap junction coupling continues to occur in dystrophic RCS rats during the early onset of retinal degeneration. Next, we investigated whether this gap junction-mediated homeostatic plasticity is modulated by the retinal circadian clock. Finally, we investigated the impact of this form of homeostatic plasticity on dystrophic RCS rats. To address these questions, we studied rod-cone gap junction coupling, the mechanism underlying this process, and its functional impact by combining tracer labeling and ERG recording in control and retinal degenerative dystrophic RCS rat retinae.

## Materials and methods

### Neural retina preparation

All experimental and animal handling procedures were performed in compliance with the Association for Research in Vision and Ophthalmology Statement and reviewed and approved by the Faculty Committee on the Use of Live Animals in Teaching and Research, Third Military Medical University. RCS (RCS-rdy-) rats and their congenic controls (RCS-rdy+) were used as control groups. Rats in these two groups (aged P10, P14, P20, P25, and P35) were tested for the ratio of gap junction coupling between the neurons in the outer plexiform layer (OPL) such as rod, cone. The animals were housed with a 12 h light–12 h dark cycle (with lights on at 6 A.M. and off at 6 P.M.). The experimental procedures were conducted in constant darkness (≤0.0001 lux). The experimenters using infrared night-vision goggles to visualize the experiments. RCS and control rats were deeply anesthetized using a mixture of ketamine (70 mg/kg) and xylazine (7 mg/kg) after a 3 h dark adaptation period. They were then enucleated, and the anterior of each eyeball was removed and placed into an extracellular bath solution that was continuously bubbled with 95% O_2_–5% CO_2_ and had the following composition per liter (1): 8.8 g of Ames' Medium (Sigma; Cat# A1420), 1.9 g of NaHCO_3_, and 7 mL of penicillin-streptomycin (Sigma; Cat# P0781). Osmolarity was then adjusted to ~280 mOsm. The retina was placed flat on the front of a piece of filter paper (Millipore, Cat#AABP02500) with the photoreceptor side up.

### Cut-loading

The protocol for cut-loading was modified from previous reports (Ribelayga et al., 2008; Choi et al., 2012). Briefly, the retinae were incubated in Ames' media containing bicarbonate and bubbled with 95% O_2_–5% CO_2_ in 6-well-plates for 30 min in the dark, as described above, with, or without test drugs. In this study, the retinae were separated among the six following groups: daytime (noon) without any drugs, nighttime (midnight) without any drugs, melatonin agonist during the daytime (2-Iodomelatonin, Tocris Cat. #0737; Delete “during the daytime”), melatonin antagonist (Luzindole, Tocris Cat. #0877) during the nighttime, melatonin receptor (MT1,2) agonist (melatonin, Tocris Cat. #3550) during the daytime, MT2 antagonist (4-P-PDOT, Tocris Cat. #1034) during the nighttime (Delete D1/D2 antagonists). We used the dyes Lucifer Yellow (0.5%, Sigma, Cat# L0259-25 MG, MW 457.25) and dextran tetramethylrhodamine (0.5%, Invitrogen, Cat# D-1817, MW 10,000) as the tracer solutions. A filter paper with a retina attached was placed on a glass slide so that the neural retina was exposed. Then, a razor blade was dipped in the tracer solution and used to make a radial cut through the retina and filter paper. The blade was held perpendicular to the retina and filter. This procedure was repeated until four cuts were made per retina. Next, filter paper was attached to the top of the neural retina, and the retina was then moved into a bubbled extracellular bath solution. After the retina was incubated for 15 min at 35°C, it was washed three times for 5 min each in a new bubbled extracellular solution (with or without drugs, depending on the first incubation conditions). The tissues were then fixed in 2% paraformaldehyde/1.5% glutaraldehyde in 0.1 M phosphate buffer (PBS, pH 7.4) for 1 h at RT(3). The retinae were washed with 0.1 M PBS overnight at 4°C and then detached from the filter paper using PBS and placed onto a slide with the photoreceptor side facing the glass.

### Imaging

After the retinae were mounted in VectaShield mounting medium, the slides were immediately observed and imaged using an Olympus laser-scanning confocal microscope. Images were recorded at a resolution of 512 × 512 pixels (635.90 × 635.90 microns) at a depth of 44 microns. The images were reloaded using Fiji software, and Z projections were used to evaluate the fluorescence of the outer neural retina or the OPL.

### Immunostaining

Enucleated eyecups were immersed in PBS containing 4% paraformaldehyde at 4°C for 2 h and then incubated in 30% sucrose at 4°C overnight. The eyecups were embedded in O.C.T. compound and cut into 10 μm-thick sections. The sections were permeabilized using 0.5% Triton X-100 for 15 min and then blocked in 5% goat serum for 1 h. Primary antibodies against AANAT (1:1,000, ab3505, Abcam), p-AANAT (phospho T29, 1:1,000, ab3439, Abcam) were diluted in blocking buffer, and the samples were incubated in this solution at 4°C overnight. The sections were rinsed three times (15 min each) with PBS at room temperature and then incubated at 37°C for 1 h with secondary antibodies. Cell nuclei were stained using 4,6-diamidino-2-phenylindole (DAPI; Invitrogen), and fluorescence images were acquired using a confocal microscope (Zeiss LSM 700, Carl Zeiss) with the same imaging parameters.

### Western blot

To perform Western blot analysis of whole retinae, the retinal samples were first lysed in ice-cold RIPA lysis buffer containing protease inhibitors (Beyotime). The lysates were centrifuged at 15,000 g at 4°C for 5 min, and the supernatants were then collected. Protein concentrations were determined using a BCA protein assay (Beyotime). A total of 25 μg of protein from each sample was loaded and electrophoresed on an SDS-polyacrylamide gradient gel (SDS-PAGE), and the blots were then transferred to PVDF membranes. The membranes were blocked in 5% non-fat milk at 37°C for 30 min. The membranes were incubated with anti-AANAT (1:1,000, ab3505, Abcam), anti-p-AANAT (phospho T29,1:1,000, ab3439, Abcam) and anti-GAPDH (1:1,000, CW0100, CWBIO) antibodies at 4°C overnight. The membranes were then rinsed with TBST and incubated with a horseradish peroxidase (HRP)-conjugated secondary antibody (Beyotime, 1:1,000) at 37°C for 1 h. The blotted proteins were visualized using an ECL detection system (Thermo Scientific).

### Electroretinogram (ERG)

Dark-adapted animals were placed under dim red light to prepare them for recording. Anesthesia was performed as described (Weymouth and Vingrys, 2008). The pupils were dilated by placing one drop each of tropicamide and phenylephrine into the eye. Corneal ERG responses were simultaneously recorded from both eyes using gold wire loops. The reference electrode was inserted under the scleral conjunctiva, and the ground electrode was placed in the tail. Amplification, stimulus presentation, and data acquisition were performed using a Reti-scan system (Roland, Germany). The following stimulus intensities were delivered at an inter-flash interval of 120 s: −40, −35, −30, −25, −20, −15, −10, −5, 0 db (0.0003, 0.00095, 0.003, 0.0095, 0.03, 0.095, 0.3, 0.95, and 3.0 cd·s·m^−2^, respectively).

### Data analysis

The cut-loading images were rotated so that the dye perfused from left to right, and the cut lines were vertical. Rectangular ROIs were chosen to cover the perfusion areas. ROI of OPL peak mainly contains photoreceptor and horizontal cells (Supplement Figure 2). We used the Plot Profile function in Fiji to obtain the gray value distance curve, and the curves were transformed for use in Igor Pro software. Using Igor Pro 6.0 (WaveMetrics Inc.), the relative fluorescence intensity was obtained by dividing each raw fluorescence value by the maximum fluorescence value, and these data were used to draw the relative fluorescence intensity distance curves. The curve fittings were created using the equation *Y* = *Y*_0_ + *Y*_max_^*^ exp^(−*x*/λ)^, where *Y* is the relative fluorescence intensity, *Y*_0_ is the background fluorescence, *Y*_*max*_ is the maximum relative fluorescence, λ is the space (length) constant, and *x* is the distance from the cut (Ribelayga et al., 2008; Choi et al., 2012). The space constant (λ) values were compared among groups and times using *t*-tests or ANOVA.

The ERG data were transferred to Igor Pro 6.2 (WaveMetrics Inc.) for further analysis. For demonstration purposes, the amplitudes of the a-waves were plotted inversely. First, the response-intensity was fitted using the following hyperbolic function (Fulton and Rushton, 1978; Hood and Birch, 1992):

$$f(V)=V_{max}{}^{*}I(I+S),$$

where *V* and *V*_*max*_ are amplitudes that were measured using a light stimulus of intensity *I* or a super-saturating stimulus, respectively, and *S* is the semi-saturation constant, which is the stimulus intensity needed to elicit a response of half-maximal amplitude. The ratio of the b-wave to the a-wave (b/a-wave ratio) was calculated using raw data and fitting data.

## Results

### Modulation of rod-cone gap junction coupling in dystrophic RCS rats

**Figure 1:**
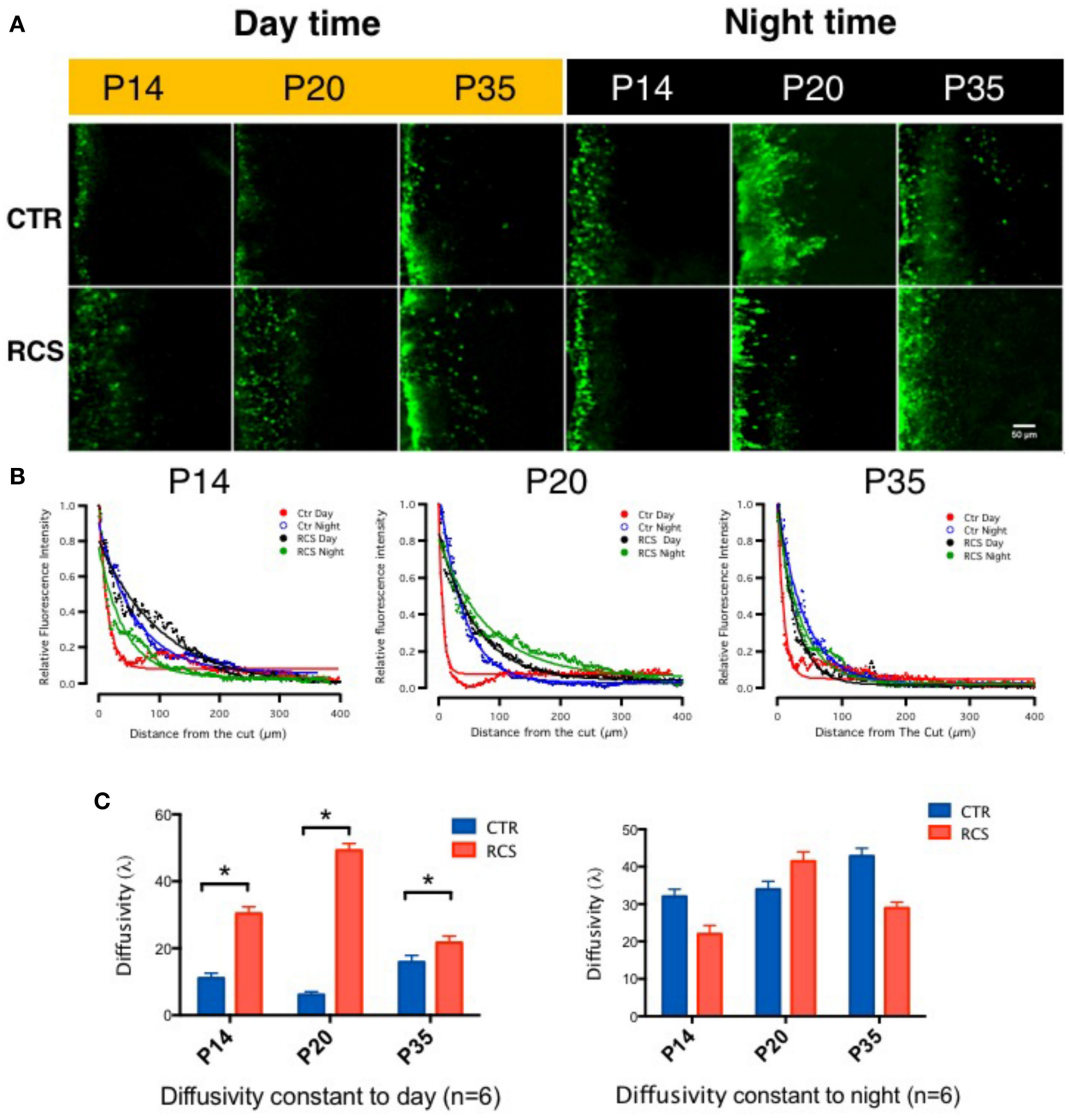
**Tracing couplings between photoreceptors in the rat retina. (A)** In the control groups, in the outer plexiform layer, the gap junction tracer Lucifer yellow was highly diffused at night (dark label) but not during the day (yellow label) under the same conditions. From P14 to P35, its diffusion capacity increased during both daytime and nighttime in these groups. These data suggest that the gap junction coupling ratio increases at night and decreases during the day and that it increases with age. In dystrophic RCS rats, the diffusion of Lucifer yellow was as extensive at night as during the day and did not increase with age. These data suggest that the gap junction coupling ratio during the daytime and in young pups is higher in dystrophic RCS rats than in controls. **(B)** The differences in relative fluorescence intensity between the two groups at P14, P20, and P35. The distance in normalized relative fluorescent intensity indicates the gap junction coupling ratio in the OPL in the rat retina. The curvature of the fitting curve (solid line) represents the diffusion capacity under different conditions (the space constant, λ). **(C)** The difference in the space constant between the daytime and nighttime in control and dystrophic RCS rats. During the daytime, diffusivity was lower in the control group than in the RCS group, and this difference was significant at P14, P20, and P35. In the RCS group, the diffusion capacity during the day increased at P14, was much higher at P20, and then dropped at P35. At night, both the control and RCS groups exhibited high diffusion capacity. The space constant increased over time in the control group but dropped at P35 in the dystrophic RCS rats. In the control rats, the diffusion constants at P14 were 11.0590 ± 3.439 (day) and 32.054 ± 4.290 (night), at P20 were 6.1062 ± 2.108 (day) and 33.980 ± 4.765 (night), and at P35 were 15.8910 ± 3.485 (day) and 42.840 ± 4.703 (night). In contrast, in the dystrophic RCS rats, the diffusion constants at P14 were 30.360 ± 4.540 (day) and 21.986 ± 4.170 (night), at P20 were 49.210 ± 4.78 (day) and 41.465 ± 5.640 (night), and at P35 were 21.686 ± 3.245 (day) and 28.980 ± 4.548 (night). ^*^P ≤ 0.05.

To measure gap junction coupling in photoreceptors, Lucifer Yellow (MW 457.25), and dextran tetramethylrhodamine (MW 10,000) were loaded during the daytime (noon) and nighttime (midnight, constant darkness) following 1 h of dark adaptation along the cut edge of flat-mounted retinae obtained from both control and dystrophic RCS rats. The red dye dextran tetramethylrhodamine is a large molecule and was used to display the cut line, whereas the small molecule Lucifer Yellow can diffuse through opened gap junctions and was used to visualize coupled cells (Supplement Figure 1C). In the control rats, the diffusion constants at P14 were 11.0590 ± 3.439 (day) (Choi et al., 2012) and 32.054 ± 4.290 (night), at P20 were 10.62 ± 2.108 (day) and 33.980 ± 4.765 (night), and at P35 were 15.8910 ± 3.485 (day) and 42.840 ± 4.703 (night). Gap junction coupling in rat photoreceptor cells is modulated by the circadian clock (Supplement Figure 1A); this was confirmed by injection of Lucifer Yellow into single photoreceptor under patching pipette (Supplement Figure 1B) which is consistent with previous results in other species (Zhang and Wu, 2004). To our surprise, we found that in retinal degeneration dystrophic RCS rats, gap junctions remained open during both day and night. In these rats, the diffusion constants at P14 were 30.360 ± 4.540 (day) and 21.986 ± 4.170 (night), at P20 were 49.210 ± 4.78 (day) and 41.465 ± 5.640 (night), and at P35 were 21.686 ± 3.245 (day) and 28.980 ± 4.548 (night) (Figure 1). For rod-cone coupling dominates in photoreceptor cells (Ribelayga et al., 2008), we calculate the diffusion in the OPL as rod-cone gap junction coupling. Thus, in this rat model, rod-cone gap junctions are functionally coupled during both daytime and nighttime, suggesting that rod-cone gap junction coupling is not regulated by the circadian clock in dystrophic RCS rats.

### Phosphorylation of the melatonin synthesis enzyme AANAT is regulated by the circadian clock

**Figure 2:**
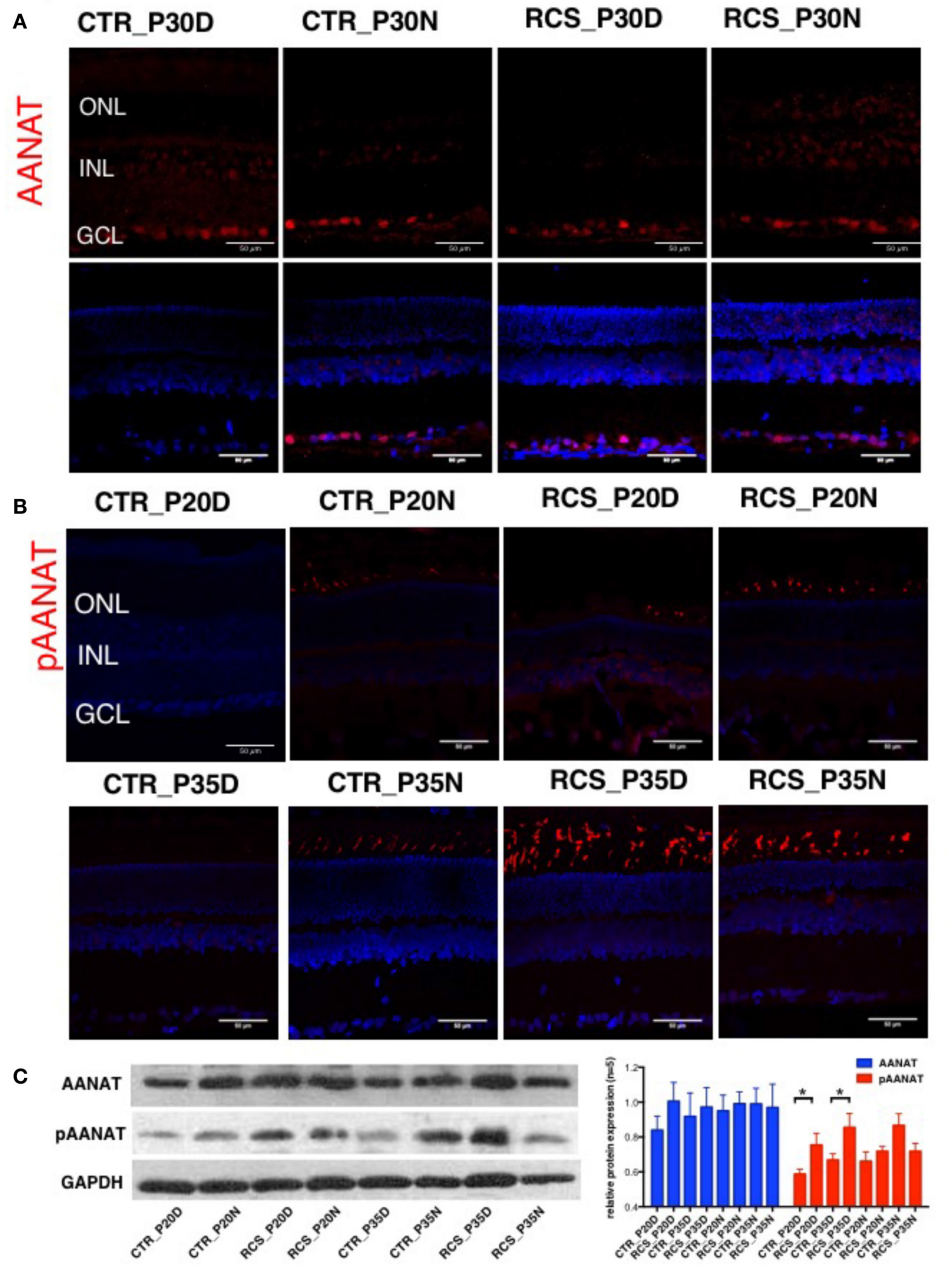
**Expression of AANAT and pAANAT in the rats' retina. (A,B)** Immunostaining for AANAT and pAANAT in the retinae of control and dystrophic RCS rats. Cell nuclei were stained using DAPI. ONL, outer nuclear layer; INL, inner nuclear layer; GCL, ganglion cell layer. **(C)** Western blot analysis for pAANAT, AANAT in the retinae of control and dystrophic RCS rats. GAPDH was used as a loading control. The relative protein expression of pAANAT and AANAT is the mean gray value of pAANAT, AANAT/value of GAPDH. Strong relative protein expression was observed in photoreceptor cells at night but not during the day in control rats **(B)**, and high relative protein expression of pAANAT was observed correspondingly **(C)**. Many strongly pAANAT-immunoreactive photoreceptors were observed during both the day and night in dystrophic RCS rats **(B)**, and high pAANAT relative protein expression in **(C)**. ^*^P ≤ 0.05.

Melatonin synthesis occurs in a rhythmic pattern that modulates rod-cone gap junction coupling (Day et al., 2010; Sandu et al., 2011; Hiragaki et al., 2014), and this process is controlled by the rate-limiting enzyme arylalkylamine N-acetyltransferase (AANAT; Sakamoto and Ishida, 1998; Pozdeyev et al., 2006; Kashiwagi et al., 2013). AANAT is activated by PKC-mediated phosphorylation at Thr29 (Choi et al., 2004). To determine the protein expression and distribution patterns of AANAT in the rat retina, we performed immunostaining in the retina using the following two AANAT antibodies: a AANAT antibody and a phosphorylation-specific (pAANAT) antibody. AANAT immunoreactivity was observed at moderate levels in ganglion cells and at low levels in photoreceptor cells (Figure 2A). Western blot analysis showed that the retinae of different groups of rats showed similar AANAT expression levels (Figure 2C). AANAT must be phosphorylated to be activated and to synthesize melatonin. In control rats, pAANAT immunoreactivity was observed in photoreceptor cells at night but not during the day (Figure 2B). In contrast, many strongly pAANAT-immunoreactive photoreceptors were observed during both the day and night in dystrophic RCS rats (Figure 2B). These data suggest that in the photoreceptor cells of normal rats, while the total amount of AANAT remains consistent, its phosphorylation is regulated by the circadian clock, allowing the cells to synthesize more melatonin at night. However, in dystrophic RCS rats, the levels of pAANAT in photoreceptor cells is high during both daytime and nighttime (RCS_P20D vs. CTR_P20D *P* = 0.049, RCS_P35D vs. CTR_P35D *P* = 0.012; Figure 2C), suggesting that photoreceptors synthesize high levels of melatonin during the day. Thus, rod-cone gap junction coupling is regulated by melatonin in a circadian clock-dependent manner.

### The signaling pathway underlying rod-cone gap junction coupling

**Figure 3:**
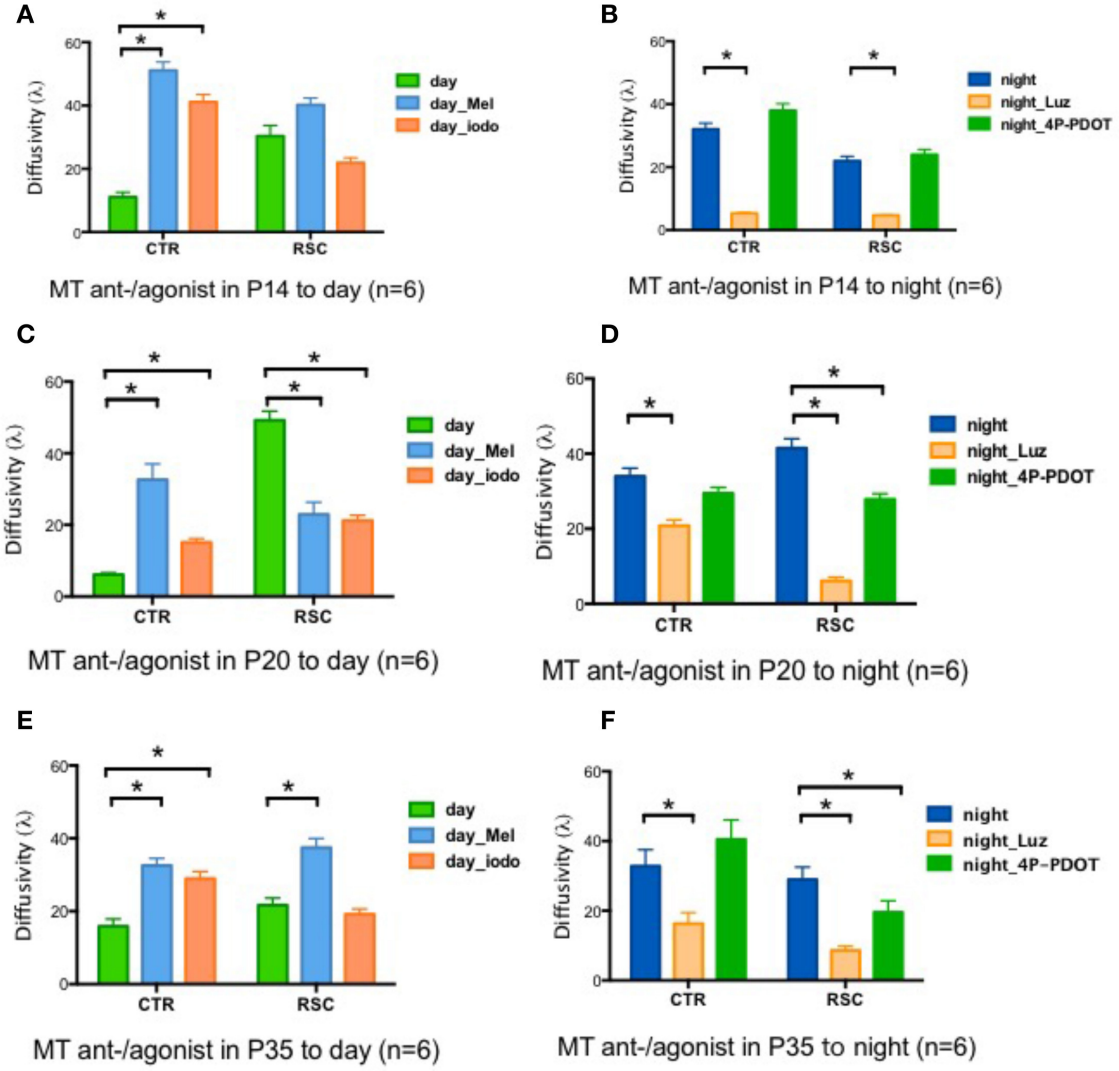
**Changes in retinal diffusivity (λ) in response to treatment with MT agonists or antagonists**. During the daytime, Iodo, and Mel increased gap junction coupling in the control groups at P14, P20, and P35 **(A,C,E)** but had the opposite effect in the RCS group at P20 **(C)**. In RCS rats, Iodo and Mel did not affect gap junction coupling at P14 **(A)**, and Mel increased diffusivity at P35d **(E)**. At night, Luz reduced diffusivity in both the control and RCS groups at P14, P20, and P35 **(B,D,F)**, and 4-P-PDOT decreased the coupling ratio in RCS rats at P20 and P35 **(D,F)** but not at P14 **(B)**. Mel, melatonin; Iodo, 2-Iodomelatonin (agonist of melatonin receptors); Luz, Luzindole (antagonist of melatonin receptors); 4-P-PDOT, Melatonin receptor 2 antagonist. ^*^P ≤ 0.05.

Two melatonin receptors (MTs) are expressed in the rat retina, namely MT1 and MT2(Fujieda et al., 1999; Sallinen et al., 2005). We performed pharmacological experiments to identify the signaling cascade by which melatonin affects gap junction coupling. Applying the MT agonists Melatonin (Mel) and 2-Iodomelatonin (Iodo) (an MT1/2 agonist) resulted in significantly higher levels of gap junction coupling in the control animals in all age groups (P14, P20, and P35) during the day (Figures 3A,C,E). In dystrophic RCS rats, the results were more complicated. Mel and Iodo had no effect on P14 dystrophic RCS rats but decreased gap junction coupling at P20 (Figures 3A,C). At P35, Mel further increased gap junction coupling, but Iodo did not (Figure 3E). Hence, during the day, MTs are not activated in control animals but are activated in dystrophic RCS rats, indicating that melatonin levels are higher in the dystrophic RCS rat than in the control animals. The MT1/2 antagonist Luz dramatically decreased gap junction coupling at night in both groups of animals (Figures 3B,D,F). The MT antagonist 4-P-PDOT, which is >300-fold more selective for MT2, had almost no effect on gap junction coupling, suggesting that MT1 is the predominant receptor involved in regulating gap junction coupling. In many species, MT1 is expressed in a variety of retinal cells, including horizontal cells, glycinergic amacrine cells, dopaminergic amacrine cells, and some ganglion cells (Savaskan et al., 2002; Sallinen et al., 2005; Sengupta et al., 2011). Rod-cone gap junction coupling is inhibited by dopamine, which is secreted from dopaminergic amacrine cells in a circadian fashion (Krizaj et al., 1998; Kothmann et al., 2011; Li et al., 2011; Choi et al., 2012; Jin et al., 2015). Thus, at night, increased extracellular melatonin binds the MT1 of dopaminergic amacrine cells to inhibit the synthesis and secretion of dopamine (Nguyen-Legros et al., 1996). Moreover, Ribelayga et al. (Ribelayga et al., 2008; Choi et al., 2012) demonstrated that the circadian clock in the retina decreases rod-cone gap junction coupling in the day by activating D2-like receptors. Taken together, these results indicate that in control animals at night and in dystrophic RCS rats at all times, the circadian clock modulates an increase in extracellular melatonin, which activates MT1 on dopaminergic cells. This leads to a decrease in the release of dopamine, resulting in the inactivation of the D2-like receptors on photoreceptors and a consequent increase in gap junction coupling.

### In dystrophic RCS rats, daytime ERG showed partial compensation by rod-cone gap junction coupling

**Figure 4:**
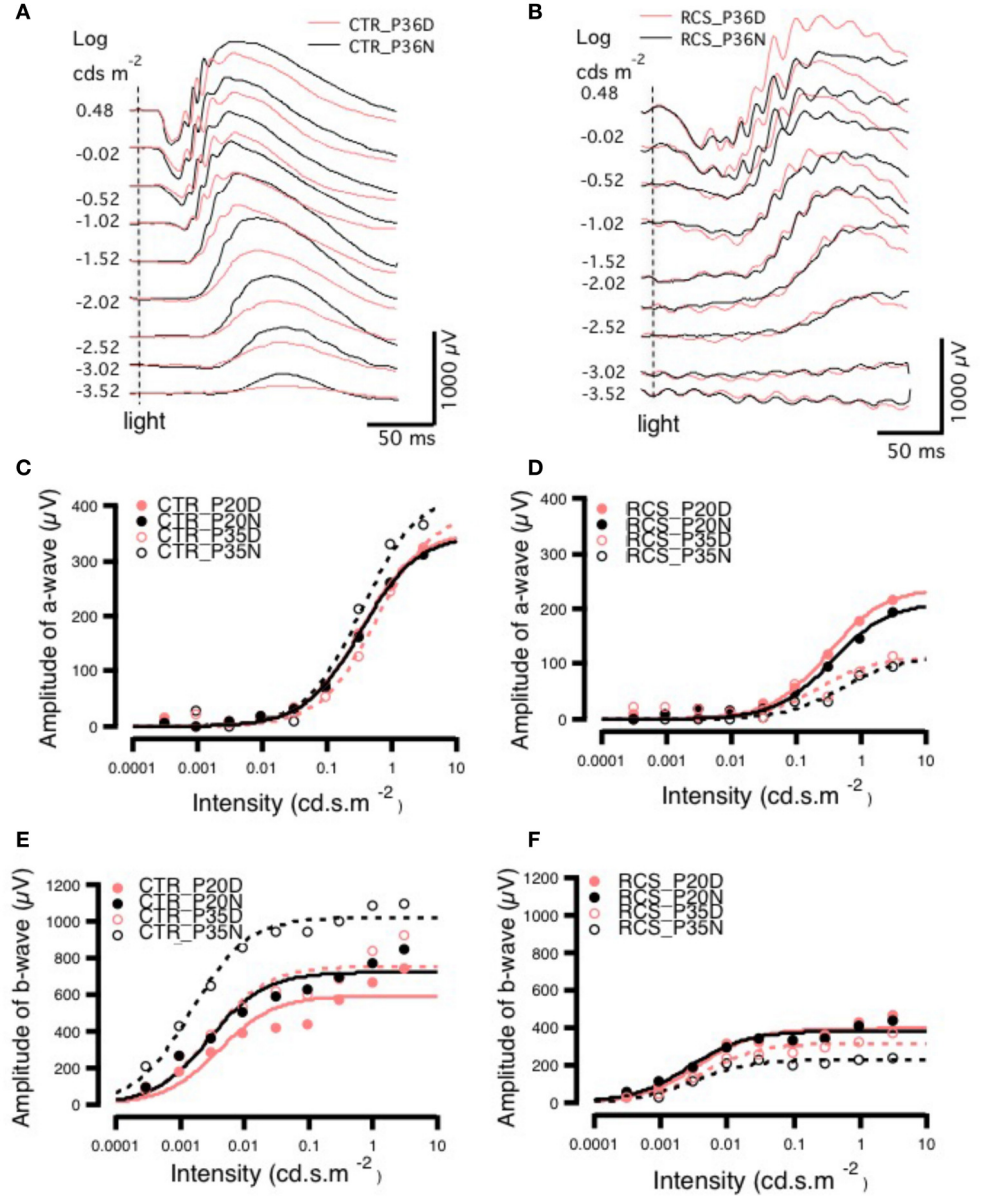
**Day-night differences in ERG a- and b-waves in rats. (A,B)** Differences in ERG amplitudes between daytime and nighttime at P35 in control and RCS rats exposed to different light intensities. In the control group, at P35, there was no difference in the amplitude of the a-wave between daytime and nighttime when animals were exposed to a lower-intensity light stimulus, but when a higher luminance intensity was applied during the night, a-wave amplitudes sharply increased. The amplitudes of b-waves were increased more by low than by high light intensities. In the RCS group, the a-wave was not markedly different between the different light intensities, but b-wave amplitudes were higher in response to higher light intensities. In the control group, the amplitudes of a-waves increased with increasing light intensity at both P20 and P35. In the controls, at P20, the amplitude of a-waves was not clearly different between daytime and nighttime in response to different stimulus intensities. In animals exposed to medium light intensities, the amplitudes of a-waves were lower in the P35 daytime group than in the P20 daytime or nighttime groups and, and in animals exposed to either medium or high intensity light, the amplitudes were higher in the P35 nighttime group than in the P20 daytime and nighttime groups **(C)**. In dystrophic RCS rats, at both P20 and P35, the amplitudes of a-waves increased with light intensity. The amplitudes of a-waves during the day in response to medium and high stimulus intensities were much lower at P35 than at P20. In addition, in the P35 RCS group, there were higher a-wave amplitudes during the daytime than at night **(D)**. At both P20 and P35, the amplitudes of b-waves increased with light intensity. The b-waves had higher amplitudes at P35 than at P20 for each luminance intensity **(E)**. The amplitudes of b-waves increased slightly with light intensity at both P20 and P35 in the control rats, and b-wave amplitudes were lower at P35 than at P20 for each light intensity **(F)**.

To determine whether rod-cone coupling alters retinal visual functions, ERG recordings were conducted in both control and RCS rats during the daytime and nighttime (Figure 4). The a-wave of the ERG results from the photoreceptor component absorbing light, while the b-wave is generated mainly by bipolar cells and represents the secondary visual signal that occurs after input from photoreceptors (Gurevich and Slaughter, 1993; Robson and Frishman, 1998, 2004; Green and Kapousta-Bruneau, 1999). The amplitudes of both the a-wave and the b-wave were smaller in dystrophic RCS rats than in the control group at ages P20 and P35 because of the loss of photoreceptors that was caused by apoptosis (Figure 4, Supplement Figure 4A). This finding is in agreement with the results of a previous study that used a similar approach in mice (Sengupta et al., 2011). In contrast, the dystrophic RCS rats showed weakened circadian regulation in the scotopic ERGs (Figures 4B,D,F; two-way ANOVA, *P* > 0.05). We averaged the ERG data from eight eyes obtained from four animals in each group and then extracted the mean responses for further analysis.

In the control group, there was no difference in the a-wave between day and night on P20 (Figure 4C), but there was a clear difference in the P35 group (Figures 4A,E), indicating circadian regulation. The *V*_*max*_ was larger at night than during the day (424.03 ± 0.0831 vs. 388.99 ± 18.9), whereas the *S* was bigger (0.5813 ± 0.0831 vs. 0.3444 ± 0.0706; Supplementary Table). These findings are consistent with the results of previous studies that have shown that cone responses are significantly smaller in amplitude at night. Thus, we conclude that in normal rats, ERGs can be used to monitor visual functions that are regulated by the circadian clock. In contrast, in dystrophic RCS rats, in the P20 and P35 groups, the *V*_*max*_ of the a-wave was similar between the day and night, whereas *S* was somewhat larger at night than during the day (Supplementary Table). These results indicated that rod-cone gap junctions are functionally open both at night and during the day. There was a more pronounced difference in b-waves between day and night. In control rats, b-wave amplitude and velocity were dramatically different between daytime and nighttime in both age groups (amplitude, P20: day 595.08 ± 48 vs. night 722.78 ± 36.6 and P35: day 755 ± 48.8 vs. night 1020.9 ± 23.7; S, P20: day 0.0040 ± 0.00197 vs. night 0.0029 ± 0.0010 and P35: day 0.0030 ± 0.0012 vs. night 0.0015 ± 0.0000; Supplementary Table). These data demonstrate that rod-cone gap junctions are functionally open at night but not during the day and that visual functions are continuing to develop at these ages. The thresholds for the a- and b-wave in each group were calculated by fitting the response-illumination intensity with a hyperbolic function because the typical threshold measurement was inapplicable in dystrophic RCS rats, in which the b-wave has a lower threshold than the a-wave. In both control and dystrophic RCS rats, the b-wave appeared ~2 log units earlier than the a-wave (Figures 4D,F), but the amplitude of the b-wave was significantly smaller in dystrophic RCS rats than in control animals. In the dystrophic RCS rats, the b-wave amplitude was smaller at P35 than at P20, similar to the a-wave due to photoreceptor loss (Supplement Figures 4A,B). To further compare the kinetics of the a- and b-waves across various groups, the a- and b-waves were normalized and fitted using a hyperbolic function (Fulton and Rushton, 1978; Hood and Birch, 1992).

**Figure 5:**
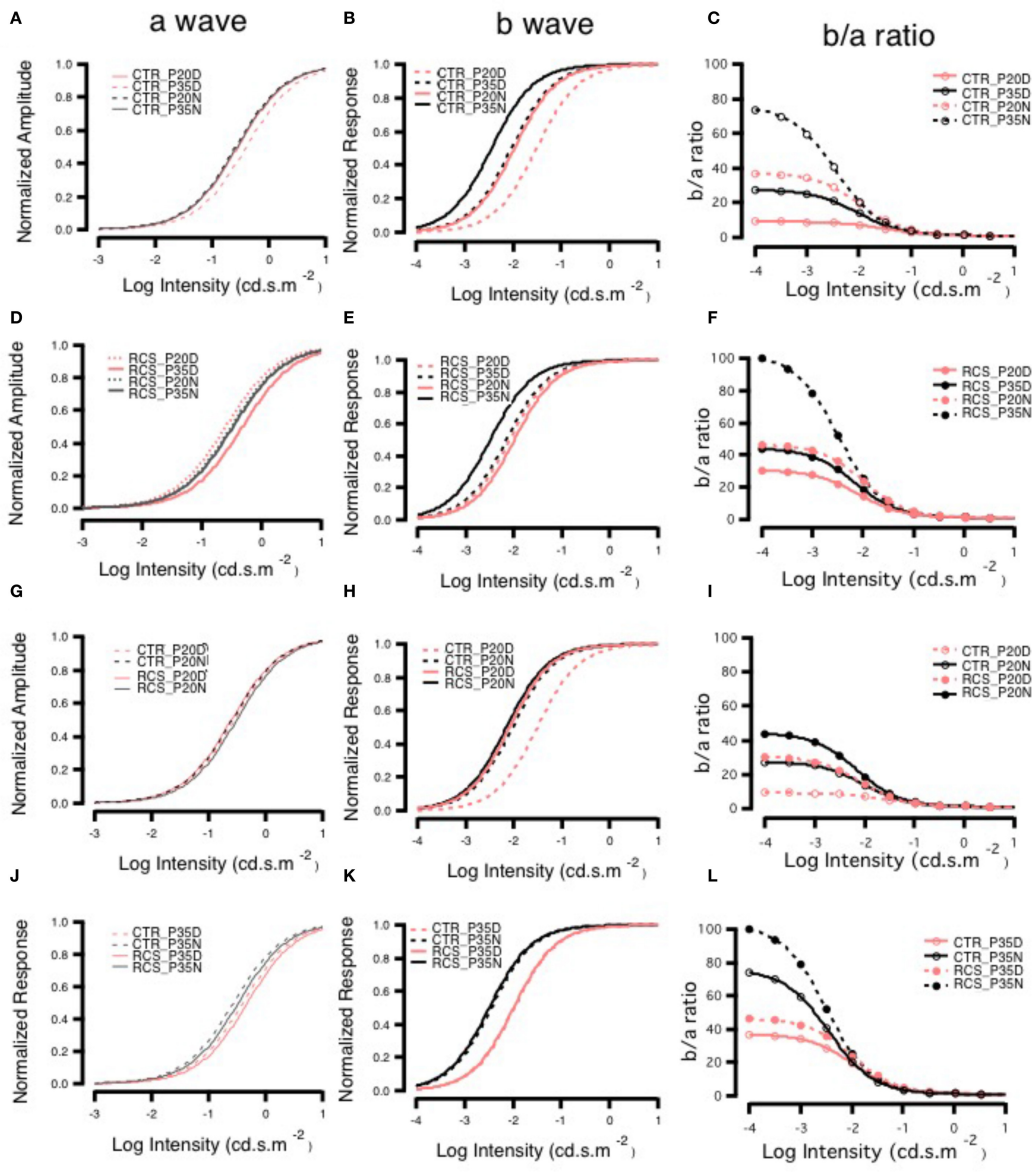
**Day-night differences in b- to a-wave ratios on ERG**. Panels **(A,D,G,J)** show the changes in the normalized amplitudes of a-waves, and panels **(B,E,H,K)** show the changes in the normalized amplitudes of b-waves. Panels **(C,F,I,L)** show the changes in the ratio of b- to a-waves, and these ratios were lower in animals exposed to higher light intensities in each group. The ratios were higher at P35 than at P20 in both control groups and higher at night than during the day in groups with the same age **(C)**. The RCS groups also had higher ratios at night than during the day. The ratios at P35 were greater than those at P20 for each light intensity. There were similar ratios in the P35 daytime and P20 nighttime groups that were exposed to different light intensities **(F)**. Panel **(I)** shows the curves of the ratios in the P20 control and RCS groups exposed to different stimulus intensities. In general, there were low gains in each group. The gains were greatest in the nighttime control group and smallest in the daytime RCS group. Panel **(L)** shows the curves of the ratios in the P35 control and RCS groups exposed to different stimulus intensities. The gains were largest in the nighttime RCS group and smallest in the daytime control group. The gains in the RCS groups were higher than those in the control groups during either the daytime or nighttime.

The a-wave reflects photoreceptor activity, while the b-wave originates from post-synaptic bipolar cells. Therefore, signal transmission in the distal retina can be transformed into the relationship between the b-wave and the a-wave. The b/a-ratio partially reflects the efficiency of signal transmission from the photoreceptors to bipolar cells. The amplitude of the a-wave grows at a faster rate than that of the b-wave, resulting in smaller b/a-ratios as intensity increases. Here, we refer to this phenomenon as *e*-gain. In this case, the b/a-ratio represents pure signal transmission efficiency because photoreceptor loss results in low amplitude a- and b-waves. The b/a-ratio therefore represents pure signal transmission efficiency and is not influenced by this side-effect. Thus, the b/a-ratio for each group was calculated using the fitting parameters for the individual group and was strongly dependent upon stimulus intensity. In control rats, *e*-gain was larger at night than during the day, a result that was confirmed by the b/a-wave ratio that was calculated from the raw data (Supplement Figure 3). Moreover, *e*-gain seemed to increase from P20 to P35, reflecting the functional maturation occurring in the connections between photoreceptors and bipolar cells during this period (Figure 5, Supplement Figure 3). This finding is also applicable to dystrophic RCS rats. Comparing the different age groups, *e*-gain values were higher in the dystrophic RCS rats in both age groups than in the comparable control groups, especially during in the daytime (Figure 5, Supplement Figures 3, 4C). Taken together, these data indicate that in dystrophic RCS rats, an increase in *e*-gain (b/a-wave ratio) compensates, at least partially, for the loss of visual input to bipolar cells that is caused by the loss of rods during early degenerative stages in these mice.

## Discussion

It has been previously demonstrated that rod-cone gap junctions open at night but not during the day in many animals, including goldfish and mice (Völgyi et al., 2013; Jin and Ribelayga, 2016). Patch-clamp data have shown that at night, dark-adapted cones can respond to light in the scotopic range because of the opening of rod-cone gap junctions (Szikra et al., 2014). Our data reveal that rats possess a mechanism of rod-cone gap junction coupling that is similar to that observed in other species (Asteriti et al., 2014). Furthermore, in the dystrophic RCS rat, which undergoes retinal degeneration, rod-cone gap junctions remain open during the day at a level that is normally observed only at night. Interestingly, although the expression of the AANAT mRNA is regulated by the circadian clock (Tosini et al., 2006), we found that there was no difference in its protein level according to time of day, but there was a difference in the levels of its phosphorylated form between night and day.

We used full-field flash ERG to determine whether gross retinal function is regulated by a circadian clock in these animals (Sengupta et al., 2011; Iuvone, 2014). Despite the fact that it is less sensitive than patch clamp, ERG can be used to monitor functional changes that are regulated by circadian clocks in control animals (Huang et al., 2012). The functional change in the a-wave is less pronounced than that in the b-wave. The b/a-ratio was calculated using raw data and fitting parameters (Sergeev et al., 2010; Ding et al., 2011; Young et al., 2012). Interestingly, in control rats, the b/a-ratio was greater at night than during the day and increased from P20 to P35. These data suggest that the amplitude of the b-wave (mainly reflecting signaling from bipolar cells) is more sensitive because the synaptic strength or efficiency of signaling between the photoreceptors and bipolar cells is regulated by a circadian clock (Emran et al., 2010; Lavoie et al., 2010; McMahon et al., 2014). The details of the mechanism underlying this phenomenon remains unknown. To our surprise, in both age groups, the b/a-ratio was larger in dystrophic RCS rats than in the control animals, although the amplitudes of the a- and b-waves were dramatically lower in dystrophic RCS rats at all intensities. While rod-cone coupling contributes to enhancing the signal of the first-order cells, there also seems to be a further effect from the synaptic connection between photoreceptor and bipolar cells and perhaps the signaling of the bipolar cell as well (Ribelayga et al., 2002).

Second, in both normal rats and dystrophic RCS rats, increased rod-cone gap junction coupling at night tunes the retina, allowing it to detect large, dimly lit objects (Tsukamoto et al., 2001; Ribelayga et al., 2008). During the day, because the number of rod cells decreases during P20–P35 in dystrophic RCS rats, the visual signal conveyed by cones can also propagate to rods via gap junction couplings. Thus, rods converge onto rod bipolar cells that also receive cone input, and this convergence increases the visual signal of bipolar cells. This homeostatic plasticity partially rescues the impaired visual functions caused by the loss of photoreceptor cells (Gargini et al., 2007; Strettoi et al., 2010).

Finally, cone survival may depend on the gap junction-mediated diffusion of nutrients and protective factors from healthy rods (Striedinger et al., 2005). In dystrophic RCS rats, rods die earlier than cones. In early stages, this process might be slowed by the diffusion of nutrients and protective factors from neighboring cones. However, cones might also die as a result of the diffusion of apoptotic factors from coupled dying rods (Ripps, 2002). When all rods are gone, the cones may die quickly as a result of the loss of support from rods. In addition, injecting melatonin increases light-induced damage to the retina, and this effect can be rescued by pretreatment with luzindole, a competitive antagonist of the melatonin receptor (Sugawara et al., 1998). The mechanism by which melatonin increases susceptibility to light damage remains unclear, but gap junction coupling is a potential explanation. In any case, higher levels of melatonin increase both susceptibility to light damage and the diffusion of apoptotic factors via gap junction couplings, and these processes in turn quicken the loss of photoreceptors.

In summary, the lifespan of cone cells is shortened because they both support rods via rod-cone gap junction couplings and act as sensors on their own. On the one hand, increasing rod-cone gap junction coupling enhances visual signals, allowing rats to detect large, dim objects at night, and this process might also allow better vision during the day if the rod bipolar pathway from cones to rods is activated. On the other hand, the pathological increase in melatonin and constant presence of open rod-cone gap junction couplings collectively hasten the death of photoreceptors because they promote the diffusion of apoptotic factors through open gap junctions.

## Author contributions

BH and YF contributed to the conception and design of the study, the collection and assembly of data, data analysis and interpretation, and manuscript writing; CW and WL contribute to the collection and assembly of data; CZ contributed to the conception and design of the study, data analysis and interpretation, manuscript writing, and the final approval of manuscript; and ZY contributed to the conception and design of the study, its financial support, data analysis and interpretation, and the final approval of manuscript.

## Funding

This work was supported by National Basic Research Program of China (973 Program, 2013CB967002), the National Natural Science Foundation Project 81130017, and 81371032.

### Conflict of interest statement

The authors declare that the research was conducted in the absence of any commercial or financial relationships that could be construed as a potential conflict of interest.
