# Supplementary material for: Homeostatic Plasticity Mediated by Rod-Cone Gap Junction Coupling in Retinal Degenerative Dystrophic RCS Rats
Source: Front Cell Neurosci. 2017 Apr 20;11:98. doi: 10.3389/fncel.2017.00098 (PMC5397418; doi:10.3389/fncel.2017.00098)
Supplement: Supplementary file 1 [file DataSheet1.docx]

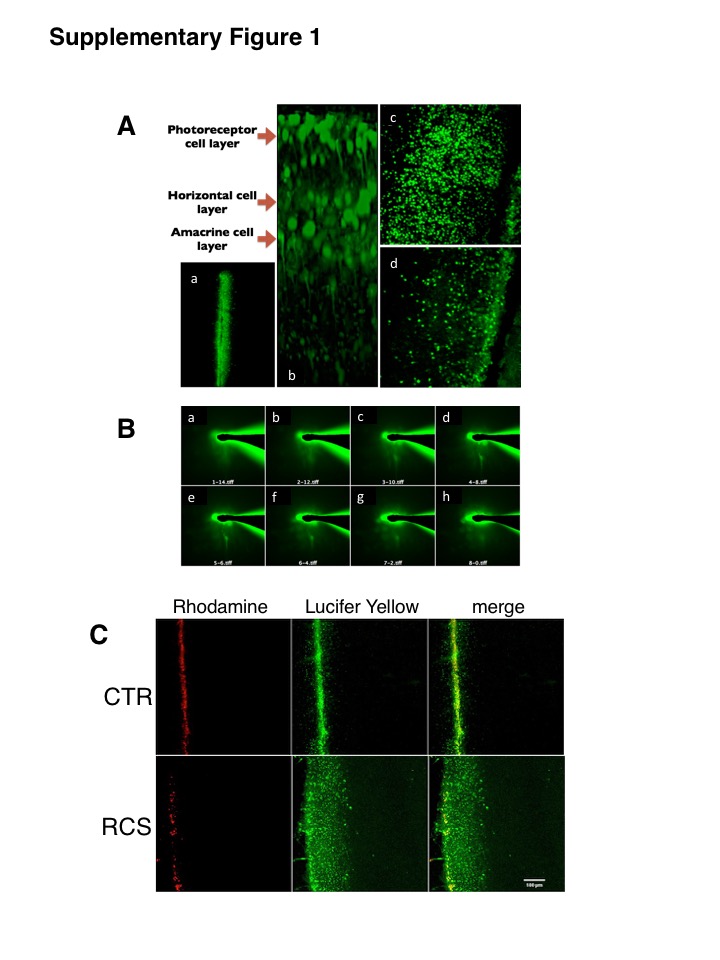


**Supplement Figure 1 Diffusion of Lucifer Yellow through gap junctions**

**A** Diffusion through gap junctions by Lucifer Yellow (MW 457.25). a. Lucifer Yellow diffuses along both sides of the incision through gup junction after cut-loading. b. Z-axis density profiling for flat mount retina and monitor the distribution of dye of different cell layers including OPL, ONL, IPL. Almost all kinds of neurons in retina(photoreceptors, horizontal cells, AII amacrine cells and αganglion cells) connect with gap junction, and are stained by LY. The different diffusion capacity of fluorescence in the control rat retinae in nighttime(c.) and daytime(d.). B Lucifer Yellow injected into single photoreceptor cell of control rat under patching pipette to monitor the gap junction coupling in photoreceptors at night. The time lapse images were taken at 2 minutes’ interval showing the dye diffused among photoreceptor cells through opening gap junction during night.  C The different diffusion of fluorescence in the control and RCS rat retinae in daytime following cut-loading with a solution of Lucifer Yellow and Rhodamine.


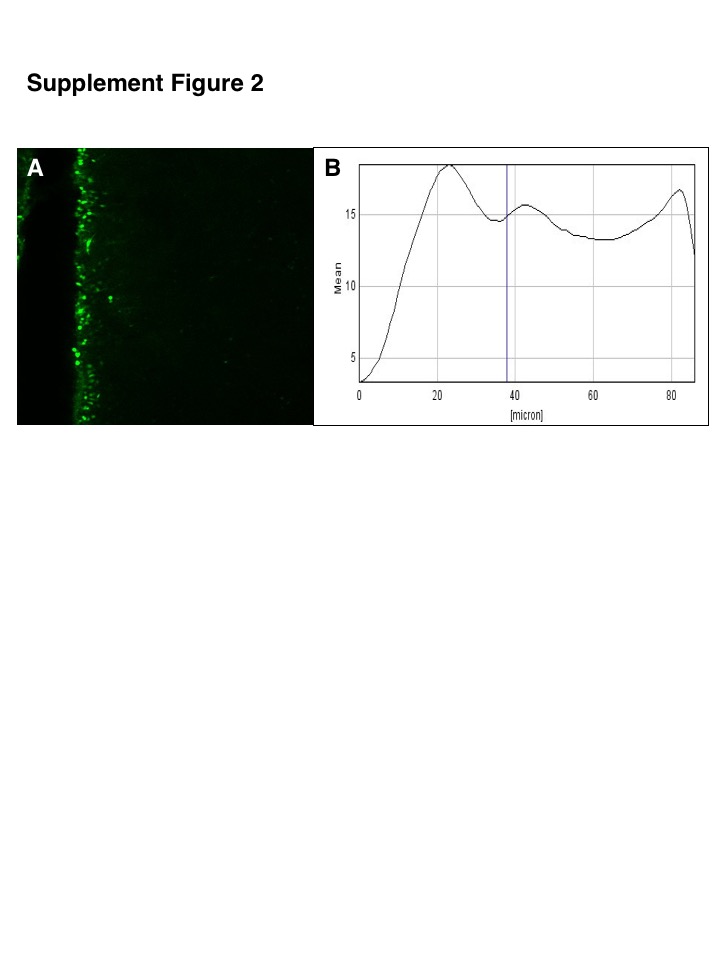


**Supplement Figure 2** **Z-axis profile of diffusion of Lucifer Yellow through gap junctions**

Under Fiji, Fiji-image-stacks-plot Z-axis profile show three peaks, the first peak indicates ganglion cell layer, and the second peak indicates IPL (amacrine cell), the third peak indicates OPL (photoreceptor, horizontal cells). The right plane of OPL (photoreceptor, horizontal cells) was chosen for further analysis.

**
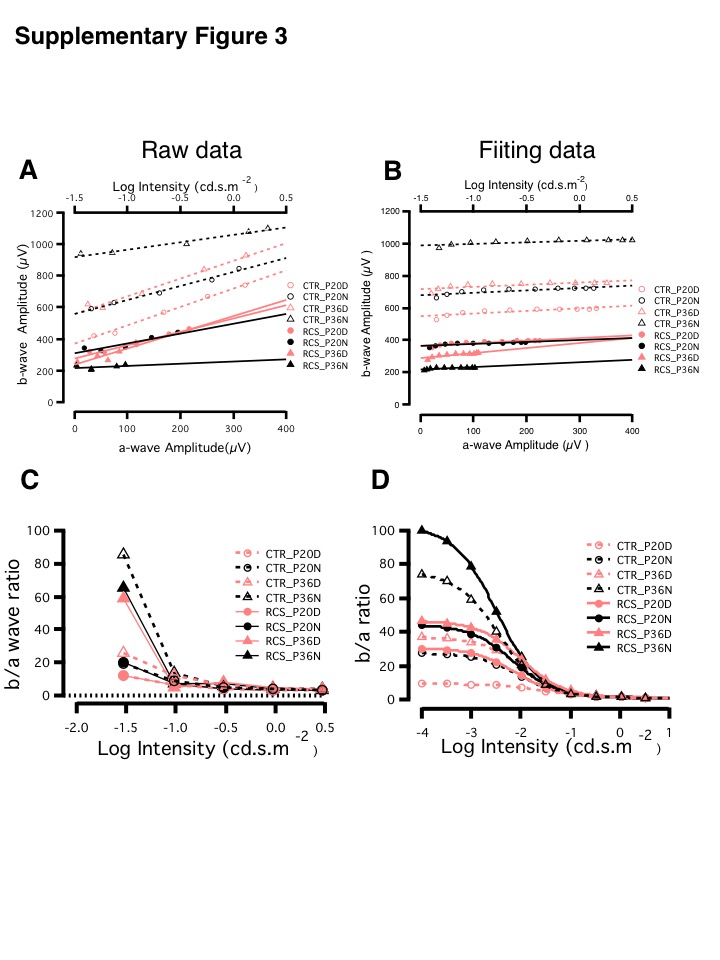
**

**Supplement Figure 3** **Day-Night Difference on ERG**

The ratios of b-wave to a-wave amplitudes in each experiment group were collectively plotted in **A** using raw data or **B** fitting data. The b/a wave ratios in each experimental group are plotted using raw data **C** or **D** fitting data.


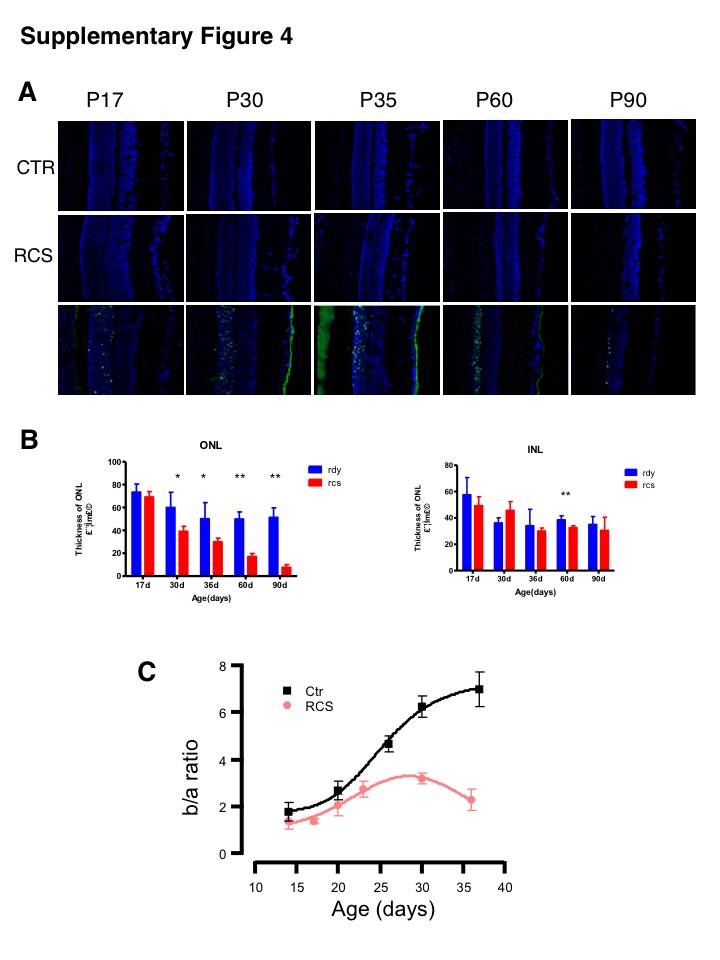


**Supplement Figure 4** **Gap junction coupling contributes to hemostatic functional regulation.** **A** Apoptotic cells (TUNEL) were observed in dystrophic RCS rats from age P17 to P90, whereas no apoptotic cells were observed in the control groups. **B** The thickness of the ONL decreased with age in dystrophic RCS rats, unlike what was observed in control animals, whereas the thickness of the INL maintained a level similar to that observed in the control animals. **C** The b/a ratio in the RCS rats was unregulated during early retinal degeneration, while photoreceptors were being lost to apoptosis. These data suggest that gap junction opening may boost the ERG b/a ratio to combat photoreceptor loss during this period.

**Supplementary table**

**Fitting data of response-intensity**


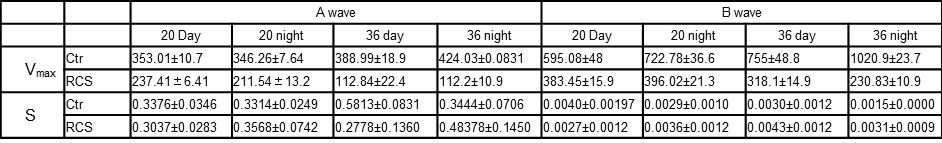


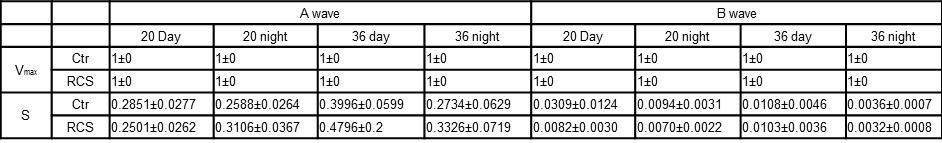
**Normalized data of fitted response-intensity**
